# Multi-omics analyses identify *HSD17B4* methylation-silencing as a predictive and response marker of HER2-positive breast cancer to HER2-directed therapy

**DOI:** 10.1038/s41598-020-72661-9

**Published:** 2020-09-23

**Authors:** Satoshi Yamashita, Naoko Hattori, Satoshi Fujii, Takeshi Yamaguchi, Masato Takahashi, Yasuo Hozumi, Takahiro Kogawa, Omar El-Omar, Yu-Yu Liu, Nobuaki Arai, Akiko Mori, Hiroko Higashimoto, Toshikazu Ushijima, Hirofumi Mukai

**Affiliations:** 1 Division of Epigenomics, National Cancer Center Research Institute, 5-1-1 Tsukiji, Chuo-ku, Tokyo, 104-0045 Japan; 2 Division of Pathology, Exploratory Oncology Research and Clinical Trial Center, National Cancer Center, 6-5-1, Kashiwanoha, Kashiwa, Chiba 277-8577 Japan; 3 Department of Medical Oncology, Musashino Red Cross Hospital, 1-26-1, Kyonan, Musashino, Tokyo 180-8610 Japan; 4 Department of Breast Surgery, Hokkaido Cancer Center, National Hospital Organization, Kikusui 4-2, Shiroishi-Ku, Sapporo, 003-0806 Japan; 5 Department of Breast and Endocrine Surgery, Ibaraki Clinical Education and Training Center, Faculty of Medicine, Tsukuba University, Tsukuba, Japan; 6 Department of Breast Surgery, Ibaraki Prefectural Central Hospital, 6528 Koibuchi, Kasama, Ibaraki 309-1793 Japan; 7 Department of Breast and Medical Oncology, National Cancer Center Hospital East, 6-5-1, Kashiwanoha, Kashiwa, Chiba 277-8577 Japan; 8 H.U. Group Innovative Cancer Laboratory, H.U. Group Research Institute, 5-1-1 Tsukiji, Chuo-ku, Tokyo, 104-0045 Japan

**Keywords:** Breast cancer, Cancer epigenetics, Cancer genomics, Predictive markers

## Abstract

HER2-positive breast cancers that achieve pathological complete response (pCR) after HER2-directed therapy consistently have good survival. We previously identified *HSD17B4* methylation as a marker for pCR by methylation screening. Here, we aimed to identify a new marker by conducting a multi-omics analysis of materials prepared by laser capture microdissection, and adding 71 new samples. In the screening set (n = 36), mutations, methylation, and expression were analyzed by targeted sequencing, Infinium 450 K, and expression microarray, respectively, and 15 genes were identified as differentially expressed and eight genomic regions as differentially methylated between cancer samples with and without pCR. In a validation set (n = 47), one gene showed differential expression, and one region had differential methylation. Further, in the re-validation set (n = 55), all new samples, only *HSD17B4* methylation was significantly different. The *HSD17B4* methylation was at the transcriptional start site of its major variant, and was associated with its silencing. *HSD17B4* was highly expressed in the vast majority of human cancers, and its methylation was present only in breast cancers and one lymphoblastic leukemia cell line. A combination of estrogen receptor-negative status and *HSD17B4* methylation showed a positive predictive value of 80.0%. During HER2-directed neoadjuvant therapy, *HSD17B4* methylation was the most reliable marker to monitor response to the therapy. These results showed that *HSD17B4* methylation is a candidate predictive and response marker of HER2-positive breast cancer to HER2-directed therapy.

## Introduction

Human epidermal growth factor receptor type 2 (HER2)-positive breast cancer is a breast cancer subtype with HER2 amplification or overexpression^1,2^. Although its prognosis used to be poor, the introduction of HER2-directed therapy dramatically improved its outcomes^3–6^. Following treatment with a HER2-directed agent and chemotherapy, some patients even achieve a pathological complete response (pCR), and these patients are known to have very long disease-free survival^7–9^. Therefore, the patients who show pCR might not need to receive surgery, but such responders cannot be identified in advance without surgery. If an accurate marker for the prediction of pCR is established, it will open up the possibility that surgery can be spared among patients with HER-2 positive breast cancer.

To predict the response of HER2-positive breast cancer to a HER2-directed agent as well as to chemotherapy, multiple exploratory studies have been conducted. As imaging markers, although digital mammogram, ultrasound, and MRI showed only low negative predictive values^10^, very low tumor residual metabolism measured by ^18^F-fluorodeoxyglucose positron emission tomography showed promise as a predictor of pCR^11–13^. As molecular markers, PIK3CA mutations were shown to be associated with reduced pCR rates^14–16^. However, the difference in the pCR rates was small, and mainly observed in a hormone receptor-positive population^15^. Gene expression markers, such as PTEN, MUC4, HSP90 and β2-adrenergic receptor, and microRNA signature, have been reported, but their validity remains unclear^16–22^. We previously isolated *HSD17B4* methylation as a candidate marker for the prediction of pCR after HER2-directed therapy^23^. Although it was validated using an independent sample set, the screening was conducted using only DNA methylation, and the number of samples was relatively small (n = 67), raising a possibility that a better marker could still be identified.

In the present study, to explore the possibility of response markers other than *HSD17B4* methylation, we conducted multi-omics screening using laser capture microdissection-purified samples. This was done by adding target sequencing of 409 cancer-related genes and expression microarray analysis. After screening and validation with 83 samples, 67 of which were used in our previous study^23^, we performed re-validation for the isolated markers using 55 previously unused samples. Furthermore, we analyzed the consequences of the marker alteration, and evaluated potential clinical utility.

## Materials and methods

### Clinical samples and patient profiles

A total of 138 HER2-positve breast cancer tissue samples was collected from the patients enrolled in a neoadjuvant clinical trial, which was reported elsewhere (Supplementary Table 1)^24^. Core needle biopsy of the tumors was carried out to obtain samples before neoadjuvant therapy. HER2-positivity was defined as overexpression by immunohistochemistry and/or amplification by fluorescent in situ hybridization based upon the 2007 ASCO/CAP Guidelines on HER2 Testing in Breast Cancer. The samples were divided into a screening set (n = 36), a validation set (n = 47), and a re-validation set (n = 55). The 36 samples in the screening set contained 14 and 15 samples used for screening and validation, respectively, in our previous study^23^. The 47 samples in the validation set contained 7 and 31 samples used for screening and validation, respectively, in the previous study^23^. Seventy-one previously unused samples were obtained, and sequentially assigned to the screening (n = 7), validation (n = 9), and re-validation (n = 55) sets.

All patients initially received HER2-directed therapy, namely trastuzumab and chemotherapy involving paclitaxel, according to the Japanese guidelines of breast cancer treatment^25^, and 35 patients received subsequent chemotherapy using epirubicin plus cyclophosphamide as previously described^24^. The patients also underwent appropriate surgery according to the size and location of the primary tumor, and the response was pathologically assessed. The study protocol was approved by the National Cancer Center Ethics Committee (Approval no. 2010-250), and was registered at the UMIN Clinical Trial Registry (Registration no. UMIN000007074)^24^. All patients provided written informed consent.

Two specimens were obtained from each patient by core needle biopsy of a primary tumor before starting neoadjuvant therapy, and fixed using two different methods. One specimen was fixed with 10% neutral buffered formalin for microscopic examination using thin sections stained with H&E, while the other was fixed using the PAXgene Tissue System (Qiagen, Hilden, Germany) and embedded in low-melting paraffin for DNA/RNA extraction. Blood samples were also collected, and stored by the PAXgene Blood DNA System (Becton, Dickinson and Company, Franklin Lakes, NJ). A certified and experienced pathologist (S. F.) analyzed the surgical specimens to determine the therapeutic response, and pCR was defined as the absence of invasive and intraductal tumor cells in a breast at surgery. The definition of pCR in this study was stricter than that in the original clinical trial^24^ in which the tissue samples were obtained, because this study aimed at stratifying patients into those that require surgery versus those who do not.

Additional core needle biopsy specimens of a primary tumor before starting neoadjuvant therapy, namely 33 triple-negative breast cancer, 16 HER2-positive breast cancer, 85 ER-positive HER2-negative breast cancer specimens were collected from formalin-fixed paraffin-embedded samples of patients who were treated between 2011 and 2015 at the National Cancer Center East Hospital. This was approved by the National Cancer Center Ethics Committee (Approval no. 2017-259), and all patients provided written informed consent.

### Cancer cell purification by laser capture microdissection

Cancer cells in tissues fixed by the PAXgene Tissue System were purified by the Leica LMD7000 system (Leica, Wetzlar, Germany) using 10 slices of 10-µm sections of block. This was conducted by an experienced pathologist (S. F.).

### Cell lines and their treatment

Four HER2-positive (AU565, BT474, HCC1954, and SK-BR-3), eleven triple-negative (BT20, BT549, HCC1395, HCC1937, HCC38, HS578T, MDA-MB157, MDA-MB231, MDA-MB436, MDA-MB453, and MDA-MB468), and five ER-positive HER2-negative (BT483, HCC1428, MCF7, T47D, and ZR-75-1) breast cancer cell lines were purchased from the American Type Culture Collection (Manassas, VA). Human Mammary Epithelial Cells (HMECs) were purchased from Cambrex (East Rutherford, NJ). A non-tumorigenic epithelial cell line, MCF10A, was purchased from the American Type Culture Collection.

For 5-aza-2′-deoxycytidine (5-aza-dC; Sigma-Aldrich, St. Louis, MO) treatment, BT20 cells whose *HSD17B4* was methylated were seeded at a density of 1 × 10^5^ cells per 10-cm plate on day 0, and were treated on days 1 and 3 as previously described^26^. The concentration of 5-aza-dC was adjusted to 0, 0.1, 0.3, 1, and 3 µM, respectively, and the cells were collected on day 5.

### Extraction of DNA and RNA

Genomic DNA was extracted by a PAXgene DNA kit (Qiagen) from tissue samples and by a PAXgene Blood DNA kit (Qiagen) from blood samples, and quantified using a Quant-iT PicoGreen dsDNA Assay Kit (Thermo Fisher Scientific, Waltham, MA). Total RNA was extracted by a PAXgene RNA kit (Qiagen) from tissue samples and by Isogen (Nippon Gene, Tokyo, Japan) from cell lines.

### Mutation analysis by targeted sequencing

Multiplex PCRs amplifying a total of 15,991 regions in 409 cancer-related genes were performed in four tubes on genomic DNA using an Ion AmpliSeq Library Kit 2.0 with the Comprehensive Cancer Panel (Thermo Fisher Scientific), as previously described^27^. The synthesized library was loaded onto an Ion PI Chip v3 (Thermo Fisher Scientific), and sequenced by an Ion Proton sequencer (Thermo Fisher Scientific) and an Ion PI Hi-Q Sequencing Kit (Thermo Fisher Scientific). Sequences were aligned onto the human reference genome hg19 with Torrent Suite v5 (Thermo Fisher Scientific). By using CLC Genomics Workbench 8.5 (Qiagen), a variant was considered a functional mutation when (i) its frequency in cancer samples was > 10%, (ii) its frequency in normal samples was < 1% (iii) its homopolymer length was < 3, (iv) it was found in both strands in 5 reads or more, (v) its coverage was > 100 reads, and (vi) it caused an amino acid change or splicing abnormality.

### Copy number alteration analysis by targeted sequencing

Copy number alterations were analyzed by VarScan 2 software^28^ using the number of reads obtained by targeted sequencing of matched tumor-normal pairs. The average reading depth of a target region in a sample was normalized by total reads. We defined -fold changes > 2 as gain, and < 0.5 as loss in this study.

### Gene expression analysis

A genome-wide gene expression analysis was conducted using an Agilent SurePrint G3 Human GE microarray 8 × 60 K array that interrogated 42,405 probes (Agilent Technologies, Santa Clara, CA) as previously described with some modifications^29^. Cy3-labeled cRNA was synthesized using the Ovation RNA Amplification System V2 (NuGEN Technologies, Redwood City, CA) and a Low Input Quick Amp Labeling Kit (Agilent Technologies), and hybridized to the microarray. The microarray was scanned using an Agilent G2565BA Microarray Scanner (Agilent Technologies). The obtained signals were processed by Feature Extraction Ver.10 (Agilent Technologies) and analyzed by GeneSpring Ver.13 (Agilent Technologies). The signal intensity of each probe was normalized such that the 75th percentile of signal intensity of all probes would be 0.

A gene-specific expression analysis was conducted by real-time RT-PCR using cDNA samples, specific primers (Supplementary Table 2), SYBR Green, and CFX Connect Real-Time PCR Detection System (Bio-Rad, Hercules, CA) as previously described with some modifications^26^. cDNA was synthesized from total RNA using SuperScript III Reverse Transcriptase (Thermo Fisher Scientific). The copy number of a target gene in a sample was measured by comparing its amplification to those of the control samples with known copy numbers. The measured copy number of a target gene was normalized to that of *GAPDH*.

### DNA methylation analysis

A genome-wide screening of differentially methylated CpG sites was conducted using an Infinium HumanMethylation450 BeadChip array (Infinium 450 K) that interrogated 482,421 CpG sites (Illumina, San Diego, CA). The raw data were normalized using MACON, a web tool for the Infinium methylation BeadArray, as described previously^30^. The methylation level of each CpG site was obtained as a β-value, which ranged from 0 (completely unmethylated) to 1 (completely methylated).

Gene-specific DNA methylation was analyzed by bisulfite pyrosequencing using the PyroMark Q96 system (Qiagen) as previously described^23^. Briefly, 200 ng genomic DNA was treated with sodium bisulfite, and eluted into 50 µl elution buffer using an innuCONVERT Bisulfite Basic Kit (Analytik Jena AG, Jena, Germany). The PCR primers for pyrosequencing are listed in Supplementary Table 2.

### Statistical analysis

Fisher’s exact test and the Mann–Whitney *U* test were used to evaluate the difference in characteristics between pCR and non-pCR. The Wilcoxon signed-rank test was used to evaluate the difference in characteristics of samples before and under treatment. All statistical analysis was conducted by R software.

### Ethics approval and consent to participate

The study was performed according to ethics approval and consent. The study protocol was approved by the National Cancer Center Ethics Committee (Approval no. 2010-250), and was registered at the UMIN Clinical Trial Registry (Registration no. UMIN000007074). The study was performed in accordance with the Declaration of Helsinki.

### Consent for publication

Informed consent for publication was obtained from all participants.

## Results

### Mutation and copy number alteration analysis did not identify any candidate marker genes

**Figure 1:**
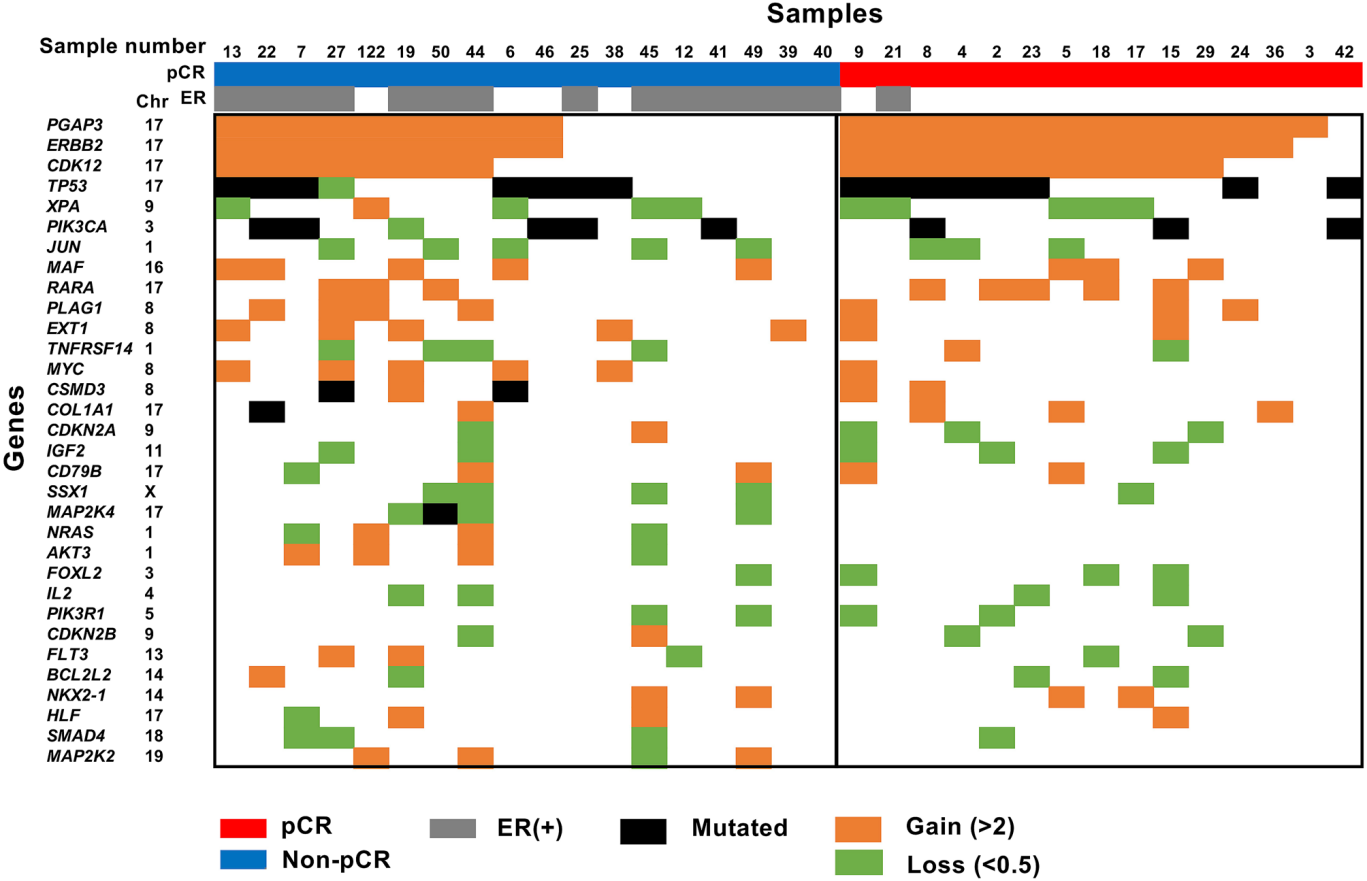
Genomic screening of response marker for HER2-positive breast cancers. Mutations and copy number alterations were analyzed and profiled in the screening set, including 15 samples with pCR and 18 samples with non-pCR. The vertical line shows the name of the genes, and ordered by frequency of alteration. When a sample had a mutation in one gene, the box is colored in black. When a sample had a gain and loss in one gene, the box is colored in orange and green, respectively. *ERBB2* (*HER2*) gain was associated with pCR. ER-negative status was also associated with pCR, which was in line with previous reports^36,37^.

As the first layer of multi-omics analysis, we conducted targeted sequencing of 409 cancer-related genes for 33 of the 36 samples in the screening set, for which both cancer and normal samples were available. A total of 85 somatic mutations were identified in the 33 cancer samples (Supplementary Table 3). *TP53* and *PIK3CA* mutation was detected in 15 and 8, respectively, of the 33 samples, which was in line with previous reports^15^. Six of the 8 *PIK3CA* mutations were at p.His1047, a well-known gain-of-function mutation^31^. However, the presence of any mutations was not associated with pCR (Fig. 1).

At the same time, copy number alterations were analyzed using coverage data of the targeted sequencing, and combined with the mutation data (Supplementary Fig. 1). Some samples showed copy number gains of *HER2*, and the presence of *HER2* gains tended to be associated with pCR (*P* = 0.07; Fig. 1). Differences in mutation frequencies of *MAP2K4*, *NRAS*, *AKT3*, and *MAP2K2* amplifications were not significant (*P* = 0.11, 0.11, 0.11, 0.11). The other genes had only a low frequency of genetic alteration, and no significant differences were present between the samples with and without pCR.

### Gene expression analysis did not identify any candidate marker genes

**Figure 2:**
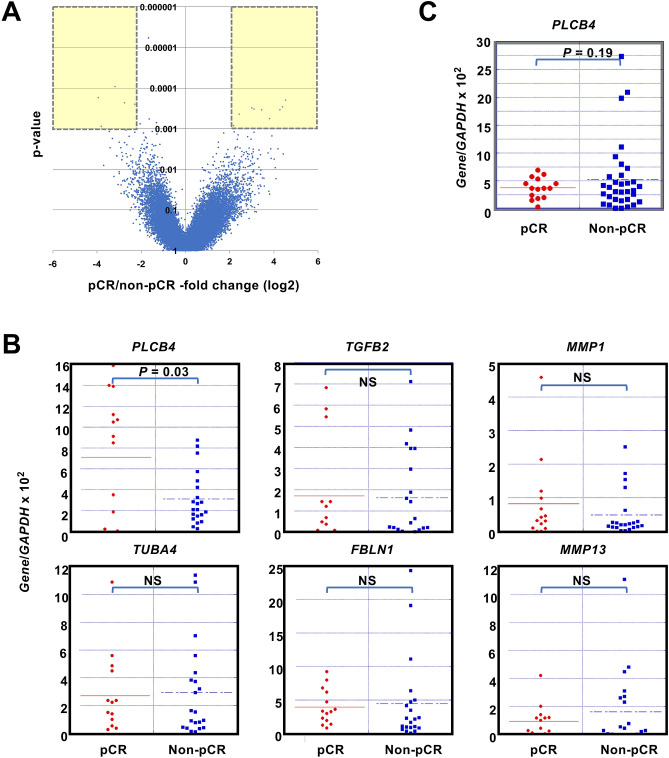
Transcriptomic screening, validation and re-validation of response marker for HER2-positive breast cancers. (**A**) Volcano plot of the results of expression microarray analysis of the screening set, including 12 samples with pCR and 19 samples with non-pCR. As differentially expressed genes between the samples with pCR and non-pCR, 15 genes were isolated (-fold changes > 4, and p values < 0.001). Among the 15 genes, 12 genes abundantly expressed (Supplementary Table 4) were further analyzed. (**B**) Expression levels of 6 of the 12 genes measured by real-time RT-PCR in the validation set. The remaining 6 genes are shown in Supplementary Fig. 2. Differential expression of only *PLCB4* was validated (*P* = 0.03). (**C**) Expression levels of *PLCB4* in the re-validation set. Differential expression of *PLCB4* was not re-validated (*P* = 0.19).

As the second layer of multi-omics analysis, we conducted expression microarray analyses of 31 of the 36 cancer samples in the screening set, for which high-quality RNA samples were available (Supplementary Table 1). A volcano plot showed that 10 and 5 genes had significantly higher and lower expression in samples with pCR than those with non-pCR, respectively (Fig. 2A, Supplementary Table 4, *P* < 0.001, fold changes > 4).

To validate the association, expression of 12 genes abundantly expressed in the screening set (average intensity > 0.5) were analyzed by real-time RT-PCR in 47 independent samples (validation set) (pCR = 17, non-pCR = 30) (Supplementary Table 1). Only *PLCB4* showed a significantly higher expression level in the samples with pCR than in those with non-pCR (*P* = 0.03) (Fig. 2B, and Supplementary Fig. 2). The association was further re-validated in 55 additional previously unused samples (re-validation set) (pCR = 17, non-pCR = 38). However, *PLCB4* expression levels did not show a significant difference between the two groups (*P* = 0.19) (Fig. 2C). Therefore, there were no re-validated marker genes whose expression levels were associated with pCR.

### DNA methylation analysis identified *HSD17B4* as a strong candidate marker gene

**Figure 3:**
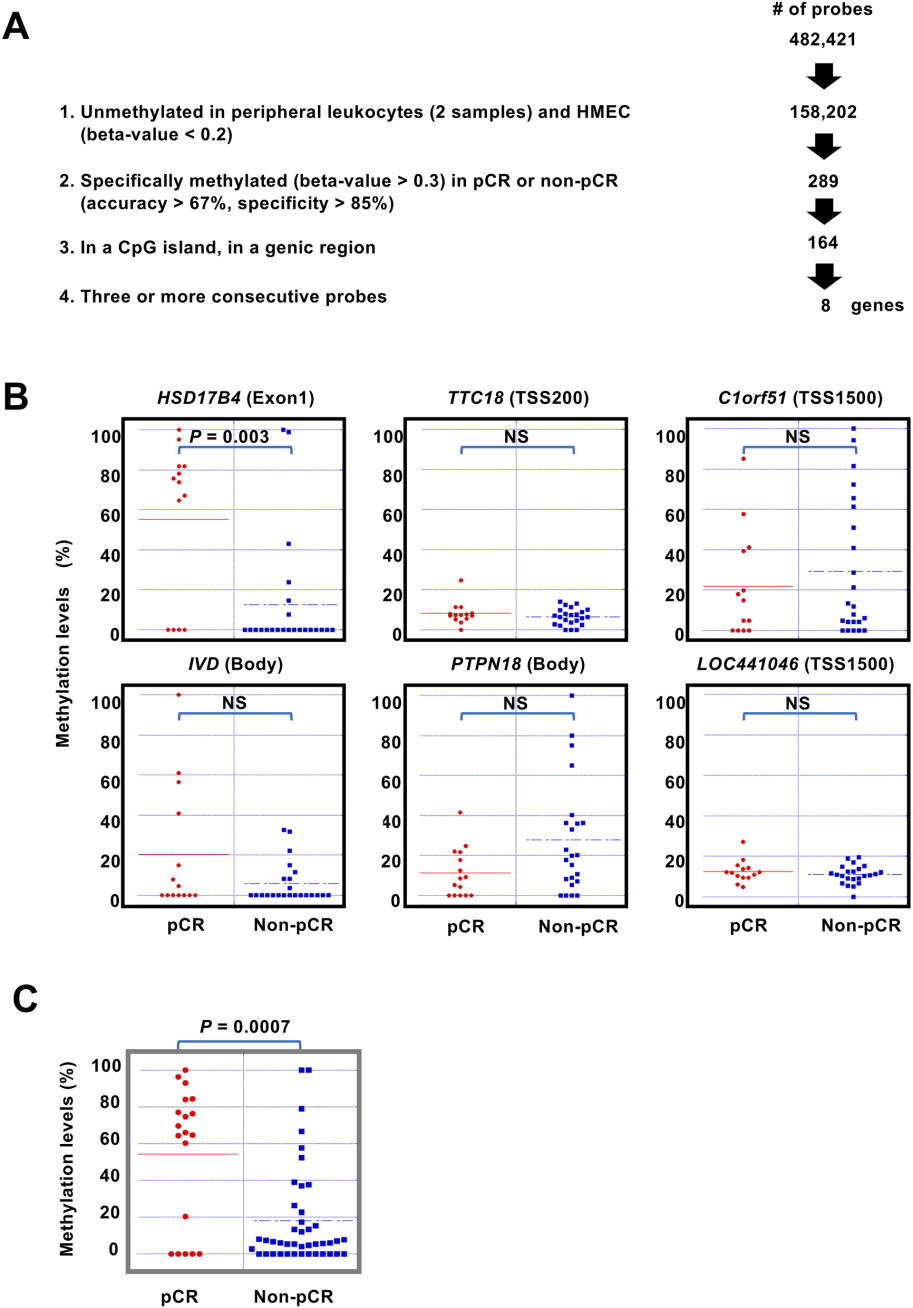
Epigenomic screening, validation and re-validation of response marker for HER2-positive breast cancers. (**A**) Workflow of the screening by DNA methylation analysis of the screening set, including 14 samples with pCR and 20 samples with non-pCR. Details are explained in the text. (**B**) Methylation levels of 6 genes measured by pyrosequencing in the validation set. Differential methylation of only *HSD17B4* was validated (*P* = 0.003). (**C**) Methylation levels of *HSD17B4* in the re-validation set. Differential methylation of *HSD17B4* was re-validated (*P* = 0.0007).

As the third layer of multi-omics analysis, we conducted Infinium 450 K beadarray analysis, which covered 482,421 CpG sites, of 34 of the 36 samples in the screening set, for which sufficient amounts of genomic DNA were available. First, to isolate normally unmethylated CpG sites, we selected 158,202 unmethylated (β-value < 0.2) CpG sites in HMECs and two samples of peripheral leukocytes (Fig. 3A). Then, we searched for individual CpG sites differentially methylated between samples with pCR and non-pCR. From the 158,202 CpG sites, we isolated 289 CpG sites hypermethylated (β-value > 0.3) in samples with pCR or in those with non-pCR with an accuracy > 0.67 and a specificity > 0.85 (Fig. 3A). Among the 289 CpG sites, 164 sites were located in CpG islands or genic regions. Among the genomic regions covered by the 164 probes, eight genomic regions (genes) had three or more consecutive probes with differential methylation. All the genes were hypermethylated in samples with pCR (Fig. 3A; Supplementary Table 5). Among the eight genes, *HSD17B4*, which was identified as a marker gene in our previous report, was included^23^.

To validate the association between the methylation of the isolated genes and pCR, we analyzed the 47 samples in the validation set. We attempted to design primers for pyrosequencing for the eight genes, and successfully designed primers for six genes (*C1orf51*, *PTPN18*, *LOC441046*, *HSD17B4*, *TTC18* and *IVD*) (Supplementary Table 5). As a result of pyrosequencing of the six genes, only the *HSD17B4* methylation level showed a significant difference between the samples with pCR and non-pCR (*P* = 0.003) (Fig. 3B). The association was further confirmed in the 55 samples in the re-validation set, which did not contain any samples from our previous study^23^. *HSD17B4* methylation levels showed a significant difference between the samples with pCR and non-pCR (*P* = 0.0007) (Fig. 3C).

### *HSD17B4* methylation led to gene silencing exclusively in breast cancers

**Figure 4:**
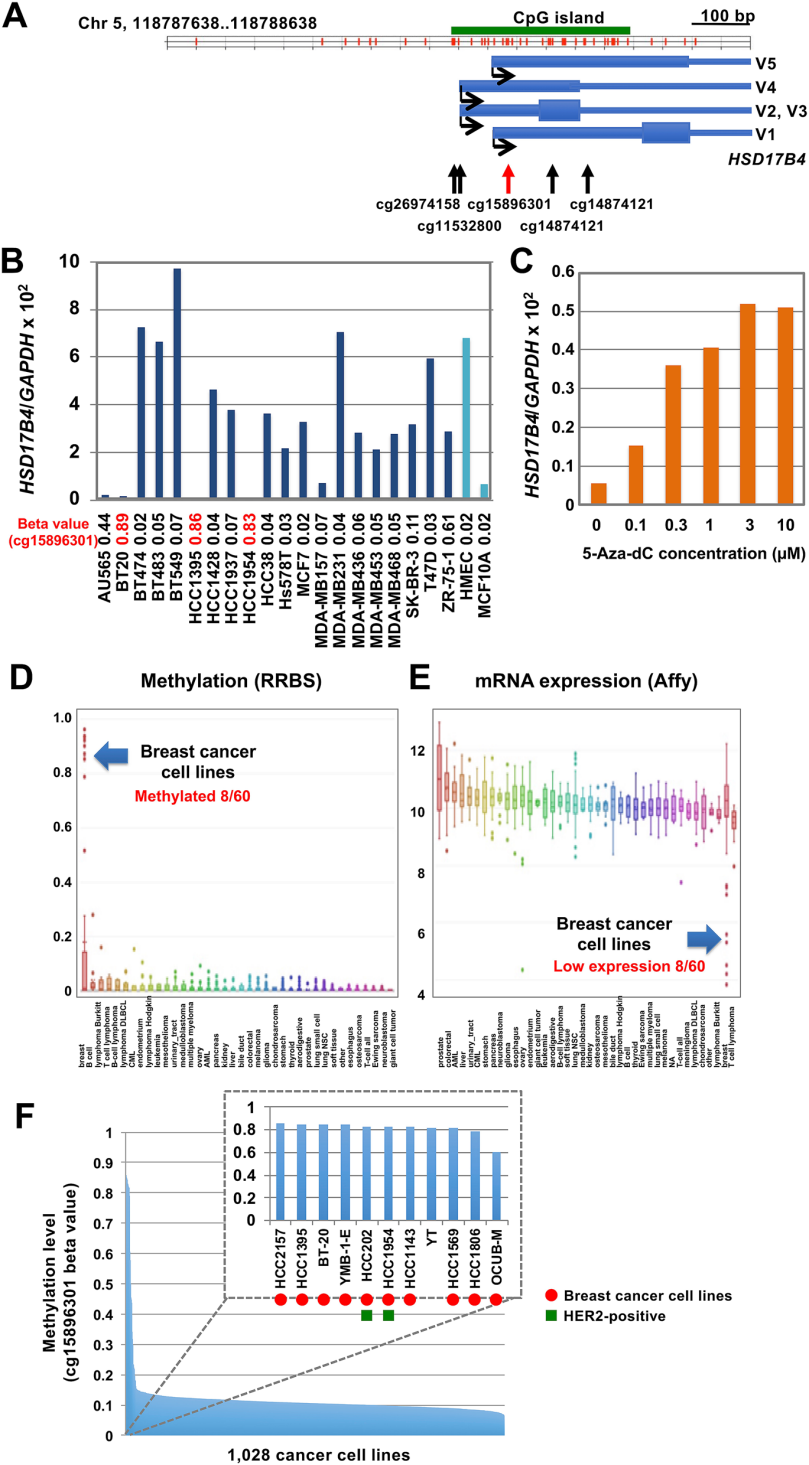
Silencing of HSD17B4 by methylation of its promoter CpG island only in specific cancer types. (**A**) Genomic structure of the promoter region of the *HSD17B4* gene. (**B**) Expression and methylation levels of *HSD17B4* in four HER2-positive (AU565, BT474, HCC1954, and SK-BR-3), eleven triple-negative (BT20, BT549, HCC1395, HCC1937, HCC38, HS578T, MDA-MB157, MDA-MB231, MDA-MB436, MDA-MB453, and MDA-MB468), and five ER-positive, HER2-negative (BT483, HCC1428, MCF7, T47D, and ZR-75-1) breast cancer cell lines. Expression levels were analyzed by real-time RT-PCR, and methylation levels were obtained by bead array analysis (high values in red, and low values in black). (**C**) Re-expression of *HSD17B4* in BT20 cells by treatment with a DNA demethylating agent, 5-aza-dC. *HSD17B4* expression was induced by the 5-aza-dC treatment in a dose-dependent manner. (**D**) Methylation levels of *HSD17B4* in cancer cell lines obtained by Cancer Cell Line Encyclopedia. Only a part of breast cancer cell lines (8/60; BT20, HCC202, HCC1143, HCC1395, HCC1569, HCC1806, HCC1954, HCC2157) showed high methylation (β-value of cg15896301>0.8). (**E**) Expression levels of *HSD17B4* gene in cancer cell lines obtained by Cancer Cell Line Encyclopedia. All of the eight breast cancer cell lines with high methylation showed low expression. (**F**) *HSD17B4* methylation in the “thousand cell line” database^35^. Its high methylation was limited to 10 breast cancer cell lines and one natural killer cell lymphoblastic leukemia cell line (YT).

The *HSD17B4* gene was known to have two transcriptional start sites (TSSs), and the marker CpG (cg15896301 probe) was located at 91 bp and 27 bp, respectively, downstream from the two TSSs (Fig. 4A). Therefore, the marker CpG site was located in exon 1, and its methylation status was expected to be associated with that of the promoter CpG island, and thus *HSD17B4* silencing. At first, to identify major transcriptional variants, expression of five known variants (V1-V5, Supplementary Fig. 3A) were analyzed, and V2 was found to be the major variant, as is known in prostate cancers^32^. However, TSSs in the DBTSS database (https://dbtss.hgc.jp/)^33^ showed that TSS of V1 (and V5) was dominant, indicating the presence of an unknown variant which has the coding region of V2 and TSS of V1. RT-PCR analyses using a downstream primer showed the presence of a novel transcript (V6, tentatively), and the transcript was the major transcript of *HSD17B4* in breast and prostate cancer cell lines (Supplementary Fig. 3B). Taken together, for the following analysis, we used universal primers that covered exons 23 to 24.

Next, we examined the association between *HSD17B4* methylation at the marker region and its loss of expression. We used 20 human breast cancer cell lines and two human breast epithelial cell lines because the effect of DNA methylation on silencing is not dependent upon subtypes. Three cell lines with high methylation at the marker CpG (two triple-negative cell lines, BT20 and HCC1395, and one HER2-positive ER-negative cell line, HCC1954) did not have *HSD17B4* expression while the other cell lines without *HSD17B4* methylation showed abundant expression (Fig. 4B). In addition, treatment of the BT20 cancer cell line with a demethylating agent, 5-aza-dC, induced its expression in a dose-dependent manner (Fig. 4C). It was reported that *HSD17B4* mRNA expression was inversely associated with *HSD17B4* methylation in HER2-positive breast cancers^34^. Therefore, we concluded that *HSD17B4* was silenced by its promoter methylation.

We also analyzed what cancer types had *HSD17B4* methylation using the Cancer Cell Line Encyclopedia (https://portals.broadinstitute.org/ccle). *HSD17B4* methylation levels were very low in almost all cancer cell lines (Fig. 4D), and its expression levels were very high in them (Fig. 4E). Only breast cancer cell lines (eight of sixty) showed high methylation levels and low expression levels. In another Infinium 450 K database of 1,028 cancer cell lines, only 10 breast cancer cell lines and one natural killer cell lymphoblastic leukemia cell line (YT) showed high methylation levels (Fig. 4F)^35^. Low expression of *HSD17B4* in the YT cells was confirmed in the GEO database (GSE53478). As for subtypes of breast cancer, the TCGA database and our analysis of 134 additional surgical specimens showed that *HSD17B4* methylation was enriched in HER2-positive and triple-negative subtypes (Supplementary Fig. 4). These results showed that *HSD17B4* was methylation-silenced solely in breast cancers and otherwise highly expressed.

### *HSD17B4* methylation was more prevalent in postmenopausal patients

To use *HSD17B4* methylation status as a predictive marker, we analyzed its independence from other factors that may affect achieving pCR using all of the samples. Negative ER status has been known to be positively associated with pCR of HER2-positive breast cancer^36,37^, and we also previously showed that combination of *HSD17B4* methylation and negative ER could predict pCR to trastuzumab and chemotherapy with high sensitivity and specificity^23^. The influence of the ER status was also observed in this study, and the positive predictive value (= pCR rate) of this combination marker was 80.0% (Supplementary Table 6). Even if a patient did not achieve pCR, the patient showed good response (≥ 1b) to the therapy (Supplementary Table 6). Importantly, when HER2-positive breast cancer patients were stratified by age, we found that *HSD17B4* methylation had a high incidence in patients older than 55 years (40.5%, Table 1). At the same time, the pCR rates, regardless of *HSD17B4* methylation, were higher in older patients (37.8%) than in younger patients (22.2%), and the predictive power of *HSD17B4* methylation (pCR rate in patients with *HSD17B4* methylation) was the same regardless of the age.

**Table 1:** Prediction of pCR of HER2-positve breast cancers using *HSD17B4* methylation, ER status, and their combination in age groups.

Age	Total	*HSD17B4* methylation			ER			Combination marker		
		High	(Positive rate)	Low	Negative	(Negative rate)	Positive	Positive	(Positive rate)	Negative
**All**										
pCR	43	30	(69.8%)	13	35	(81.4%)	8	24	(55.8%)	19
(pCR rate)	(33.6%)	(75.0%)		(14.8%)	(53.8%)		(12.7%)	(80.0%)		(19.4%)
Non-pCR	85	10	(11.8%)	75	30	(35.3%)	55	6	(7.1%)	79
Total	128	40	(31.3%)	88	65	(50.8%)	63	30	(23.4%)	98
**> 55**										
pCR	28	22	(78.6%)	6	23	(82.1%)	5	18	(64.3%)	10
(pCR rate)	(37.8%)	(73.3%)		(13.6%)	(54.8%)		(15.6%)	(78.3%)		(19.6%)
Non-pCR	46	8	(17.4%)	38	19	(41.3%)	27	5	(10.9%)	41
Total	74	30	(40.5%)	44	42	(56.8%)	32	23	(31.1%)	51
**45–55**										
pCR	9	5	(55.6%)	4	6	(66.7%)	3	3	(33.3%)	6
(pCR rate)	(33.3%)	(83.3%)		(19.0%)	(46.2%)		(21.4%)	(75.0%)		(26.1%)
Non-pCR	18	1	(5.6%)	17	7	(38.9%)	11	1	(5.6%)	17
Total	27	6	(22.2%)	21	13	(48.1%)	14	4	(14.8%)	23
**<45**										
pCR	6	3	(50.0%)	3	6	(100.0%)	0	3	(50.0%)	3
(pCR rate)	(22.2%)	(75.0%)		(13.0%)	(60.0%)		(0.0%)	(100.0%)		(12.5%)
Non-pCR	21	1	(4.8%)	20	4	(19.0%)	17	0	(0.0%)	21
Total	27	4	(14.8%)	23	10	(37.0%)	17	3	(11.1%)	24

### *HSD17B4* methylation may be a better response marker than Ki-67

**Figure 5:**
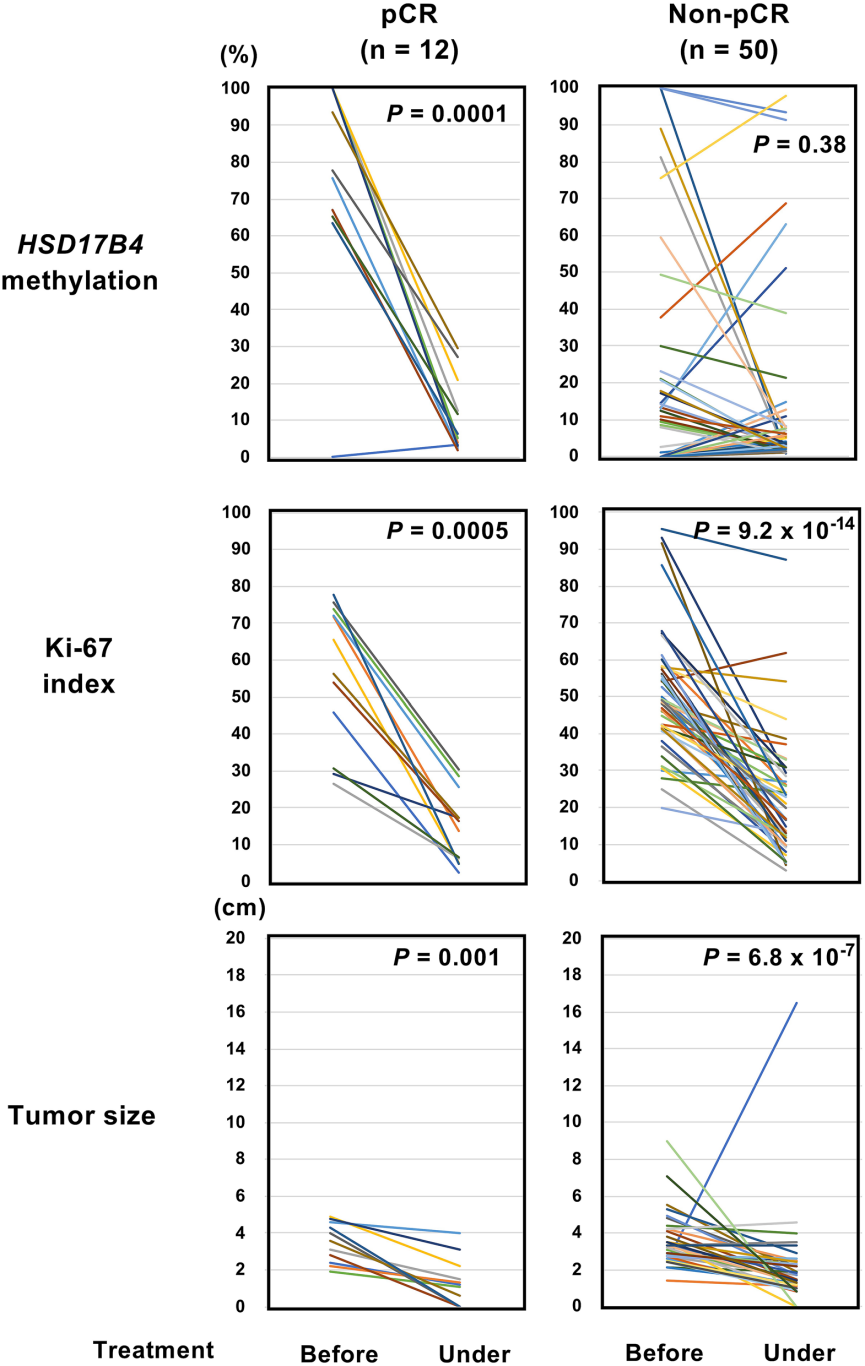
Better prediction of response during neoadjuvant therapy by HSD17B4 methylation than by Ki-67 index. *HSD17B4* methylation levels and Ki-67 index were measured by pyro-sequencing and immunohistochemistry, respectively, using biopsy specimens before and during the neoadjuvant therapy including 12 samples with pCR and 50 samples with non-pCR. The tumor size was measured by echogram. The *HSD17B4* methylation level consistently decreased only in cancer samples that achieved pCR, and was considered to be a better response marker than the Ki-67 index or tumor size.

Changes in the Ki-67 index are proposed as a useful predictor of response to preoperative therapy in breast cancer^38^. Therefore, we compared the powers of change of *HSD17B4* methylation and the Ki-67 index in cancer samples during treatment to predict a final response, namely as a response marker. The tumor size obtained by echogram was also analyzed. The *HSD17B4* methylation level consistently decreased in cancer samples that achieved pCR, while not consistently in cancer samples that did not achieve pCR (Fig. 5). Among the 15 samples that showed large decreases of the *HSD17B4* methylation level (∆β  ≥ 50%), 11 samples (73%) achieved pCR. In contrast, the Ki-67 index or the tumor size obtained by echogram decreased in most cancer samples whether they achieved pCR or not. These results showed that *HSD17B4* methylation may be a better response marker to chemotherapy than Ki-67.

## Discussion

In the present study, we identified that *HSD17B4* methylation is a candidate predictive marker of HER2-positive breast cancer to HER2-directed therapy. Compared with our previous study with 67 samples^23^, we added 71 new samples, genomic and transcriptomic screening, and purification by laser capture microdissection. Nevertheless, no new markers other than *HSD17B4* methylation were identified. The 55 samples for the re-validation were only newly collected samples. This showed that *HSD17B4* methylation was a promising marker to predict pCR of HER2-positive breast cancer to HER2-directed therapy.

*HSD17B4* methylation as predictive marker was more effective with ER negative status. The sensitivity and specificity of the combined marker in the overall three sets of samples was 55.8% and 92.9%, respectively. *HSD17B4* methylation was more prevalent in postmenopausal patients, but the predictive power of *HSD17B4* methylation was the same across all age groups. For future surgery-free treatment, patients who are predicted to be sensitive to trastuzumab and chemotherapy should respond with a high probability. Our combined marker achieved a high specificity of 92.9%, which is approaching a clinically tolerable level. To establish *HSD17B4* methylation as a predictive marker, we are now conducting a prospective study, named PASSION trial (UMIN000028065)^39^. By introducing sequential chemo-radiotherapy, we expect that the pCR rate and resultantly the specificity of the marker will further increase, and we will prospectively evaluate the predictive performance of the marker. In this trial, all patients will receive surgery as standard therapy to ensure their safety.

We also analyzed cancer samples during neoadjuvant chemotherapy for *HSD17B4* methylation, Ki-67 index and tumor size to monitor response to the therapy. Unlike Ki-67 index and tumor size*, HSD17B4* methylation decreased consistently in cancer samples that achieved pCR. This indicated that cancer cells with *HSD17B4* methylation were preferentially killed by the treatment, and that *HSD17B4* methylation may also be useful as a response marker for treatment.

Methylation of the *HSD17B4* marker CpG was found to be associated with that of its promoter CpG island, and associated with *HSD17B4* silencing. The vast majority of cancer cell lines highly express *HSD17B4*, thereby suggesting that *HSD17B4* expression is essential for the survival of most cancer cell lines. In contrast, *HSD17B4* methylation-silencing only in some breast cancer cell lines suggested that the silencing can oppositely provide a growth advantage only in breast cancer cells. Biochemically, HSD17B4 is involved in the β-oxidation of long-chain fatty acids in peroxisomes^40^ and in the conversion of 17β-estradiol into inactive estrone^41^. High *HSD17B4* expression is speculated to be essential for fatty acid metabolism. Therefore, we can speculate that impairment of β-oxidation and the resultant metabolism may lead to therapy sensitivity although promising approach to increase sensitivity of HER2-positive breast cancers to targeted therapy is proposed^42^. At the same time, *HSD17B4* silencing can be advantageous for some breast cancer cells as it is involved in estrogen catabolism.

The limitation of the study is the presence of the 67 previously used samples in the 83 screening and validation sets. This could have led to the isolation of *HSD17B4* methylation again. However, we isolated additional candidates by methylation screening at the same time, and only *HSD17B4* methylation was re-validated in the 55 new samples. Importantly, this time, we conducted multi-omics screening by adding mutation and expression screening and using laser capture dissection-purified samples. Nevertheless, only *HSD17B4* methylation was re-validated, showing its importance as a single gene marker for the sensitivity of HER2-positive breast cancers to HER2-directed therapy.

Ten of 33 breast cancers clinically diagnosed as HER2-positive did not show HER2 amplification in the molecular analysis. This was considered due to the difference between the clinical definition of “HER2-positive” and the molecular definition of “HER2 gene copy number gain”. For example, if 20% of tumor cells are HER2-positive due to a fivefold increase of HER2 copy number, this sample would be clinically "HER2-positive". However, its molecular HER2 copy number would be only a 1.8-fold increase (= 80% × 1 + 20% × 5), and this sample would not have “HER2 gene copy number gain”.

In conclusion, *HSD17B4* methylation was strongly associated with the sensitivity of HER2-positive breast cancer to HER2-directed therapy, and no additional markers were isolated. If the marker is clinically established as a predictive marker, the ultimate breast-conserving treatment without the need for surgery may become a treatment option.

## Supplementary information

Supplementary file1

## Data Availability

The data that support the findings of this study are available from the corresponding author upon reasonable request.
