# Supplementary material for: Multi-omics analyses identify HSD17B4 methylation-silencing as a predictive and response marker of HER2-positive breast cancer to HER2-directed therapy
Source: Sci Rep. 2020 Sep 23;10:15530. doi: 10.1038/s41598-020-72661-9 (PMC7511952; doi:10.1038/s41598-020-72661-9)
Supplement: Supplementary file 1 — Supplementary file1 [file 41598_2020_72661_MOESM1_ESM.pdf]

Supplementary Information

Multi-omics analyses identify *HSD17B4* methylation-silencing as a predictive and response marker of HER2-positive breast cancer to HER2-directed therapy

Satoshi Yamashita, Naoko Hattori, Satoshi Fujii, Takeshi Yamaguchi, Masato Takahashi, Yasuo Hozumi, Takahiro Kogawa, Omar El-Omar, Yu-Yu Liu, Nobuaki Arai, Akiko Mori, Hiroko Higashimoto, Toshikazu Ushijima*, and Hirofumi Mukai

*Correspondence to Toshikazu Ushijima, Division of Epigenomics, National Cancer Center Research Institute, Tokyo, Japan, 5-1-1 Tsukiji, Chuo-ku, Tokyo 104-0045, Japan; phone: +81 3 3547 5240, fax: +81 3 5565 1753, E-mail: tushijim@ncc.go.jp

Supplementary Table 1 List of samples

Sample ID	Age	Group	Useage for previous study (Fujii *et al.* 2017)	Pathological complete response<br>0:Non-pCR<br>1:pCR	Genomic screening	Transcriptome screening	Epigenome (methylation) screening	Analyzed samples during the treatment	Clinical tumor stage<br>1:T1<br>2:T2<br>3:T3<br>4:T4	Clinical nodal stage<br>1:N0<br>2:N1<br>3:N2<br>4:N3	Clinical stage<br>2:IIA<br>3:IIB<br>4:IIIA<br>5:IIIB<br>6:IIIC	Histology<br>1:Invasive ductal carsinoma<br>2:Invasive lobular carcinoma<br>3:Others	Treatment (2 course)<br>1:wPTX+Tmab<br>2:EX+Tmab	ER<br>1:Positive<br>2:Negative	PgR<br>1:Positive<br>2:Negative	Chemotherapy effect<br>1:Grade0<br>2:Grade1a<br>3:Grade1b<br>4:Grade2a<br>5:Grade2b<br>6:Grade3 (rDCIS)<br>7:Grade3
1-7C	47	Screening	Validation	0	✓	✓	✓		3	2	4	1	2	1	1	3
11-40C	71	Screening	Validation	0	✓	✓	✓		2	1	2	1	2	1	2	2
11-41C	60	Screening	Validation	0	✓	✓	✓		2	1	2	1	1	1	1	2
11-45C	52	Screening	Validation	0	✓	✓	✓		2	1	2	1	1	1	2	3
12-122C	36	Screening	Validation	0	✓		✓		2	2	3	1	1	2	2	6
2-46C	70	Screening		0	✓	✓	✓		4	3	5	1	2	2	2	2
3-13C	61	Screening	Screening	0	✓	✓	✓		4	4	6	1	1	1	2	4
3-38C	54	Screening	Validation	0	✓	✓	✓		2	1	2	1	2	2	2	5
4-27C	61	Screening	Screening	0	✓	✓	✓		2	1	2	1	1	1	1	3
5-49C	29	Screening	Validation	0	✓	✓	✓	✓	2	1	2	1	1	1	1	2
5-54C	68	Screening	Validation	0		✓	✓	✓	2	1	2	1	2	1	1	3
5-6C	58	Screening	Screening	0	✓	✓	✓		1	2	2	1	2	2	2	4
6-25C	39	Screening	Screening	0	✓	✓	✓		2	2	3	1	1	1	1	3
6-39C	63	Screening	Validation	0	✓	✓	✓		2	2	3	1	1	1	1	2
7-12C	64	Screening	Screening	0	✓	✓	✓		1	2	2	1	1	1	1	2
8-19C	39	Screening	Screening	0	✓	✓	✓		3	2	4	1	2	1	1	5
8-22C	66	Screening	Screening	0	✓	✓	✓		2	3	4	1	2	1	2	6
8-44C	51	Screening	Validation	0	✓	✓	✓		2	1	2	1	1	1	2	6
8-50C	42	Screening	Validation	0	✓	✓	✓	✓	1	2	2	1	1	1	1	2
9-43C	54	Screening	Validation	0		✓	✓		3	3	4	1	2	2	2	3
1-14C	59	Screening		1		✓	✓		2	2	3	1	1	2	2	7
1-3C	48	Screening		1	✓		✓		2	1	2	1	1	2	2	7
2-15C	67	Screening		1	✓	✓	✓		2	3	4	1	1	2	2	7
2-4C	56	Screening	Screening	1	✓		✓		3	4	6	1	1	2	2	7
3-18C	65	Screening	Screening	1	✓	✓	✓		2	1	2	1	1	2	2	7
3-2C	54	Screening		1	✓		✓		3	2	4	1	1	2	2	7
3-5C	50	Screening	Screening	1	✓	✓	✓		2	2	3	1	1	2	2	7
3-9C	49	Screening		1	✓	✓	✓		2	2	3	1	1	2	2	7
4-23C	62	Screening	Screening	1	✓	✓	✓		2	3	4	1	1	2	2	7
4-29C	34	Screening		1	✓				4	4	6	1	ND	2	1	7
5-8C	65	Screening	Validation	1	✓	✓	✓		1	2	2	1	1	2	2	7
8-17C	54	Screening	Screening	1	✓	✓	✓		2	1	2	1	1	2	2	7
8-21C	58	Screening	Screening	1	✓	✓	✓		4	2	5	3	1	1	1	7
8-36C	60	Screening	Validation	1	✓	✓			2	1	2	1	1	2	2	7
8-42C	56	Screening	Validation	1	✓	✓	✓		2	2	3	1	1	2	2	7
9-24C	68	Screening	Screening	1	✓	✓	✓		2	4	6	1	1	2	2	7
1-146C	64	Validation	Validation	0				✓	2	3	4	1	1	2	2	6
1-33C	58	Validation	Screening	0					2	1	2	1	2	1	1	3
1-58C	56	Validation	Validation	0					2	1	2	1	2	1	1	4
10-62C	64	Validation	Validation	0				✓	2	1	2	1	2	1	1	4
10-69C	72	Validation	Validation	0				✓	2	1	2	2	2	1	1	2
11-74C	62	Validation	Validation	0				✓	2	1	2	1	2	1	2	3
11-77C	59	Validation	Validation	0				✓	2	2	3	1	1	1	1	4
11-94C	59	Validation	Validation	0					4	2	5	1	1	1	2	5
12-113C	62	Validation	Validation	0				✓	3	2	4	1	2	2	2	6
12-143C	62	Validation		0					2	2	3	1	2	2	2	6
13-124C	57	Validation	Validation	0				✓	2	2	3	1	1	2	2	6
13-140C	42	Validation	Validation	0					2	2	3	1	1	2	2	6
2-37C	72	Validation	Validation	0					4	1	5	1	1	2	2	5
3-111C	61	Validation	Validation	0				✓	2	1	2	1	1	2	2	5
3-129C	58	Validation	Validation	0				✓	2	1	2	1	2	1	1	4
3-93C	29	Validation	Validation	0					3	3	4	1	1	2	2	4
4-16C	59	Validation		0				✓	2	2	3	1	1	1	2	2
4-30C	63	Validation	Screening	0					2	1	2	1	1	2	2	4
5-28C	56	Validation	Screening	0					2	2	3	1	2	2	2	4
6-11C	63	Validation	Screening	0					2	2	3	2	1	1	1	2
6-92C	54	Validation	Validation	0				✓	3	2	4	1	1	1	1	4
7-75C	72	Validation	Validation	0				✓	3	2	4	1	1	2	2	3
7-86C	45	Validation	Validation	0					2	1	2	1	1	1	1	5
8-141C	48	Validation	Validation	0				✓	2	2	3	1	2	2	2	2
8-158C	39	Validation		0				✓	2	2	3	1	1	1	1	6
8-47C	61	Validation	Validation	0				✓	2	2	3	1	2	1	1	4
8-68C	43	Validation	Validation	0				✓	2	2	3	1	2	1	1	5
8-90C	39	Validation	Validation	0				✓	2	1	2	1	1	1	1	4
8-95C	63	Validation	Validation	0					2	2	3	1	1	2	2	5
9-144C	50	Validation		0				✓	2	3	4	1	2	1	2	6
1-106C	65	Validation		1				✓	2	1	2	1	2	1	1	7
1-107C	58	Validation	Validation	1				✓	2	2	3	1	1	2	2	7
1-156C	61	Validation	Validation	1					2	2	3	1	1	2	2	7
1-98C	35	Validation	Validation	1					3	4	6	1	1	2	2	7
11-32C	64	Validation	Screening	1					2	2	3	1	1	2	2	7
11-91C	55	Validation	Validation	1				✓	3	2	4	1	1	1	2	7
12-35C	50	Validation	Validation	1					2	2	3	1	1	1	2	7
13-152C	46	Validation	Validation	1					2	2	3	1	1	2	2	7
3-130C	41	Validation	Validation	1					2	2	3	1	1	2	2	7
3-26C	49	Validation	Screening	1					2	1	2	1	1	1	1	7
4-138C	57	Validation		1					2	2	3	1	1	1	2	7
4-139C	61	Validation		1				✓	3	2	4	1	1	2	2	7
8-136C	55	Validation		1					4	2	5	2	1	2	1	7
8-20C	60	Validation	Screening	1					2	2	3	1	1	2	1	7
8-82C	63	Validation	Validation	1				✓	2	1	2	1	1	1	1	7
9-52C	67	Validation		1					3	2	4	3	2	2	2	7
9-89C	57	Validation	Validation	1				✓	2	1	2	1	1	2	2	7
10-167C	33	Revalidation		0				✓	2	1	2	1	1	1	1	4
10-225C	56	Revalidation		0				✓	2	2	3	1	2	1	1	3
10-31C	69	Revalidation		0					2	1	2	2	1	1	2	4
10-51C	68	Revalidation		0				✓	2	2	3	1	1	1	2	3
12-183C	70	Revalidation		0				✓	2	2	3	1	2	2	2	5
12-210C	67	Revalidation		0				✓	2	2	3	1	2	2	2	2
13-197C	35	Revalidation		0				✓	2	1	2	1	1	1	1	4
13-230C	39	Revalidation		0				✓	3	3	4	1	1	2	2	6
13-252C	55	Revalidation		0				✓	2	3	4	1	1	2	2	5
2-102C	71	Revalidation		0				✓	2	2	3	1	1	2	2	6
2-151C	72	Revalidation		0				✓	1	3	4	1	1	1	1	2
3-182C	38	Revalidation		0				✓	2	1	2	1	1	1	1	4
3-217C	69	Revalidation		0					3	2	4	1	1	1	2	2
3-63C	25	Revalidation		0				✓	3	2	4	1	1	1	1	5
4-190C	50	Revalidation		0				✓	2	2	3	1	1	2	2	6
4-195C	52	Revalidation		0				✓	2	1	2	1	1	1	1	6
4-227C	42	Revalidation		0				✓	3	4	6	1	1	1	1	5
5-232C	62	Revalidation		0					1	2	2	3	2	2	2	2
6-203C	46	Revalidation		0					2	2	3	1	1	2	2	6
6-34C	35	Revalidation		0					2	2	3	1	1	1	2	6
7-149C	49	Revalidation		0					2	1	2	1	1	1	1	3
7-179C	63	Revalidation		0				✓	2	2	3	1	1	2	2	4
7-189C	35	Revalidation		0				✓	2	1	2	1	1	1	1	3
7-228C	46	Revalidation		0				✓	2	1	2	1	1	1	1	1
8-181C	37	Revalidation		0					2	1	2	2	1	1	1	4
8-191C	54	Revalidation		0				✓	2	4	6	1	1	1	1	2
8-202C	55	Revalidation		0				✓	2	2	3	1	2	2	2	2
8-209C	39	Revalidation		0				✓	2	1	2	1	2	1	1	5
8-226C	66	Revalidation		0				✓	2	1	2	1	1	1	1	2
8-229C	64	Revalidation		0				✓	2	2	3	1	1	2	2	3
8-234C	51	Revalidation		0				✓	2	1	2	1	1	1	2	5
8-247C	70	Revalidation		0				✓	2	1	2	1	1	1	1	4
8-254C	40	Revalidation		0				✓	2	1	2	1	2	1	1	3
8-57C	57	Revalidation		0					2	2	3	1	1	2	1	6
8-64C	37	Revalidation		0				✓	2	1	2	1	2	1	1	4
9-170C	62	Revalidation		0				✓	2	2	3	1	1	2	2	4
9-214C	66	Revalidation		0				✓	2	2	3	1	1	1	2	4
9-250C	71	Revalidation		0					3	2	4	1	1	1	1	6
10-61C	51	Revalidation		1				✓	2	1	2	1	1	2	2	7
12-199C	75	Revalidation		1					2	2	3	1	1	2	2	7
13-198C	43	Revalidation		1					2	4	6	1	1	2	2	7
13-224C	39	Revalidation		1				✓	2	3	4	1	1	2	2	7
13-257C	44	Revalidation		1				✓	2	2	3	1	1	2	2	7
2-206C	63	Revalidation		1					2	2	3	1	1	2	2	7
3-172C	60	Revalidation		1					2	1	2	3	1	2	2	7
3-246C	63	Revalidation		1					2	4	6	1	1	1	2	7
5-166C	61	Revalidation		1				✓	2	1	2	1	1	2	2	7
6-162C	72	Revalidation		1					2	1	2	1	2	2	2	7
7-157C	55	Revalidation		1					1	2	2	1	1	2	2	7
7-256C	62	Revalidation		1					2	1	2	1	1	2	2	7
8-115C	40	Revalidation		1					2	2	3	1	1	2	2	7
8-218C	56	Revalidation		1				✓	2	2	3	1	2	1	1	7
8-238C	56	Revalidation		1					1	3	4	2	1	2	2	7
9-112C	65	Revalidation		1				✓	2	1	2	1	2	2	2	7
9-205C	56	Revalidation		1					2	2	3	1	1	2	2	7

Supplementary Table 2 List of primers

Gene symbol	Analysis	Forward primer (5'->3')	Reverse primer (5'->3')	Length (bp)
PLCB4	real-time RT-PCR	CATGCAAAGGAACACAGTACCA	CTAGGATTTTCTCATGAGTCGACTT	103
TGFB2	real-time RT-PCR	GAAGAATGCTTCCAATTTGGTG	GGACTTGAGAATCTGATATAGCTC	109
GPR110	real-time RT-PCR	ACTTCGTTGTGGTGCTGCTAGT	AGCAGAGGGGTCAGAATGAGGA	127
MMP13	real-time RT-PCR	TCCTGTTCTCAGGAAACCAG	AAACTGTATGGGTCCGTTGA	167
C6orf203	real-time RT-PCR	TGAACTCAGGCTGAATGAGG	TCCGCATAACTGTCTCTGTTC	119
MMP1	real-time RT-PCR	CCAAAATGATAGCACATGACTTTCC	CTGGAGAGTCAAAATTCTCTTCGT	140
GUCY1A3	real-time RT-PCR	AATTTGAGTCCTGCAGTGTACC	TCGAGGGGTAAACACGAAAC	95
TUBA4A	real-time RT-PCR	CAACCAGATGGTAAAGTGTGATCC	CAATGGCAGCGTTGACATCC	101
PMEPA1	real-time RT-PCR	GCACAGTGTCAGGCAACGGA	TCTCGTGCTGCAGGTACGGA	141
FBLN1	real-time RT-PCR	GAGTGGCTTTATACAAGATGCTC	CTGTGTTGATGCATGTATGCC	101
MRPL43	real-time RT-PCR	GAGGAGAGCATCCACTGCAA	CTAGGGTTGTCGGTGTGGAA	119
PDE5A	real-time RT-PCR	ACAAGAGAGCTACAGTCGTTAG	GTGCTGTTTCCAGATCAGAC	109
HSD17B4	real-time RT-PCR	AGGAAATAGGACGCCGCCTAA	CCTTGGTACACTTTTCCAGAACCA	148
GAPDH	real-time RT-PCR	GGTCGGAGTCAACGGATTT	TGATGGCAACAATATCCACTT	86
HSD17B4 V1	real-time RT-PCR	ATGGTTATTCTTGAGGCACCG	GATCTTCTCCATAGGGTTATTGC	159
HSD17B4 V2	real-time RT-PCR	AAATCGGCAAGTCACTGACCCT	CCAAATCATTCACAACAACTAACGC	240
HSD17B4 V3	real-time RT-PCR	GCAAGTCACTGACCCTCGT	CCAAATCATTCACTGCCC	180
HSD17B4 V4	real-time RT-PCR	TCGTCCCGCCCCCGCCAT	CTTCTCCATAGGGTTATTGCATAG	241
HSD17B4 V5	real-time RT-PCR	GGTCTCTCAAGCAGGATT	GTCATAATAATCTTCTGTTTCTT	393
HSD17B4 V6 (*+V2+V5*)	real-time RT-PCR	AGCGGCTCTGCTTGTTCGTGT	CCAAATCATTCACAACAACTAACGC	168
HSD17B4	Bisulfite-pyro-seq	GGTGAGTTTATGAATAAGGTTTGTAA	Bio-CACAAATCCTAACAACTCCCATTAC	268
HSD17B4	Bisulfite-pyro-seq	TAAAGATATATATAAGAATAAGTAG (sequencing)		
C1orf51	Bisulfite-pyro-seq	Hs_CG21831898_02_PM PyroMark CpG assay		
PTPN18	Bisulfite-pyro-seq	Hs_CG26797073_01_PM PyroMark CpG assay		
LOC441046	Bisulfite-pyro-seq	Hs_CG18925923_01_PM PyroMark CpG assay		
ZNF354C	Bisulfite-pyro-seq	Hs_CG12623648_03_PM PyroMark CpG assay		
TTC18	Bisulfite-pyro-seq	Hs_CG08496452_02_PM PyroMark CpG assay		
KLC2	Bisulfite-pyro-seq	Hs_CG18046087_02_PM PyroMark CpG assay		
IVD	Bisulfite-pyro-seq	Hs_CG11934304_02_PM PyroMark CpG assay		
PISD	Bisulfite-pyro-seq	Hs_CG26448406_02_PM PyroMark CpG assay		

Supplementary Table 3 List of mutations

Sample ID	Gene	Chromosome	Position	Type	CDS mutation	AA mutation	Count	Coverage	Frequency (%)
2C	*PDE4DIP*	chr1	144917875	SNV	c.1609G>C	p.Glu537Gln	152	509	29.9
2C	*TP53*	chr17	7578553	SNV	c.377A>G	p.Tyr126Cys	83	101	82.2
2C	*DCC*	chr18	50985681	SNV	c.3472G>A	p.Glu1158Lys	205	322	63.7
2C	*TET2*	chr4	106180924	SNV	c.3952G>C	p.Glu1318Gln	232	580	40.0
2C	*EZH2*	chr7	148513800	SNV	c.1481C>T	p.Pro494Leu	246	331	74.3
2C	*KAT6A*	chr8	41798467	SNV	c.2932A>T	p.Arg978Trp	42	96	43.8
2C	*NUP214*	chr9	134022931	SNV	c.2000C>T	p.Pro667Leu	429	1019	42.1
3C	*TRIP11*	chr14	92471936	SNV	c.2384T>C	p.Val795Ala	12	81	14.8
4C	*TP53*	chr17	7577538	SNV	c.743G>A	p.Arg248Gln	342	463	73.9
4C	*ROS1*	chr6	117687424	SNV	c.2627C>T	p.Ala876Val	147	924	15.9
5C	*MN1*	chr22	28196443	SNV	c.89T>G	p.Met30Arg	123	1091	11.3
6C	*TP53*	chr17	7577538	SNV	c.743G>A	p.Arg248Gln	59	339	17.4
6C	*SF3B1*	chr2	198267484	SNV	c.1873C>T	p.Arg625Cys	112	815	13.7
6C	*MAGI1*	chr3	65438931	SNV	c.1042+2T>C	Possible splice site disruption	31	127	24.4
6C	*CSMD3*	chr8	03731..11330	Deletion	c.8958_8974+8delTTTA CCAGAATGTATCAGTA	p.Pro2986fs	27	120	22.5
7C	*CDH1*	chr16	68842595	SNV	c.532-1G>T	Possible splice site disruption	184	221	83.3
7C	*TP53*	chr17	7577539	SNV	c.742C>G	p.Arg248Gly	105	138	76.1
7C	*PIK3CA*	chr3	178952084	SNV	c.3139C>T	p.His1047Tyr	129	213	60.6
8C	*TP53*	chr17	7577105	SNV	c.833C>T	p.Pro278Leu	175	374	46.8
8C	*ZNF521*	chr18	22804595	SNV	c.3287G>T	p.Cys1096Phe	97	369	26.3
8C	*DCC*	chr18	49867235	SNV	c.78T>A	p.His26Gln	192	662	29.0
8C	*PIK3CA*	chr3	178952085	SNV	c.3140A>G	p.His1047Arg	410	506	81.0
9C	*TP53*	chr17	7576897	SNV	c.949C>T	p.Gln317*	167	182	91.8
12C	*ZNF521*	chr18	22806249	SNV	c.1633G>T	p.Gly545Trp	347	1178	29.5
13C	*MLLT10*	chr10	21962744	SNV	c.1517C>G	p.Ser506*	42	354	11.9
13C	*RB1*	chr13	48916820	SNV	c.350T>C	p.Phe117Ser	11	98	11.2
13C	*TP53*	chr17	7577559	SNV	c.722C>T	p.Ser241Phe	259	712	36.4
13C	*KEAP1*	chr19	10600420	SNV	c.1435G>C	p.Asp479His	110	667	16.5
13C	*PMS1*	chr2	190742112	SNV	c.2749C>G	p.His917Asp	29	231	12.6
13C	*MYH9*	chr22	36708111	SNV	c.1711A>G	p.Ile571Val	186	482	38.6
13C	*FGFR4*	chr5	176522696	SNV	c.1793G>A	p.Arg598Gln	140	1267	11.1
13C	*PKHD1*	chr6	51889601	SNV	c.5007C>G	p.Ile1669Met	122	802	15.2
13C	*IKBKB*	chr8	42171947	SNV	c.800G>A	p.Ser267Asn	58	480	12.1
15C	*BCL11B*	chr14	99641144	SNV	c.2029C>T	p.Pro677Ser	28	93	30.1
15C	*SETD2*	chr3	47125238	SNV	c.6032A>C	p.Lys2011Thr	124	568	21.8
15C	*PIK3CA*	chr3	178916918	SNV	c.305T>A	p.Ile102Asn	88	660	13.3
15C	*APC*	chr5	112111393	SNV	c.490A>C	p.Asn164His	13	85	15.3
17C	*ASXL1*	chr20	31023718	SNV	c.3203G>A	p.Arg1068Gln	149	1407	10.6
18C	*NOTCH2*	chr1	120458246	SNV	c.7099C>T	p.Gln2367*	161	933	17.3
18C	*CBL*	chr11	119149386	SNV	c.1394A>G	p.Asp465Gly	392	1332	29.4
18C	*RNF213*	chr17	78320962	SNV	c.8827T>C	p.Cys2943Arg	385	1816	21.2
18C	*ALK*	chr2	29451867	SNV	c.2698G>A	p.Ala900Thr	355	2101	16.9
19C	*BLNK*	chr10	97976441	SNV	c.575C>T	p.Pro192Leu	35	247	14.2
19C	*IDH2*	chr15	90631908	SNV	c.445C>T	p.Arg149Trp	1833	3576	51.3
19C	*PTPRT*	chr20	41076877	SNV	c.1543G>A	p.Val515Ile	18	138	13.0
19C	*SETD2*	chr3	47165471	SNV	c.655A>G	p.Ile219Val	305	949	32.1
19C	*ROS1*	chr6	117645552	SNV	c.5584T>C	p.Phe1862Leu	31	89	34.8
21C	*TP53*	chr17	7577570	SNV	c.711G>A	p.Met237Ile	594	1829	32.5
22C	*ATM*	chr11	108163384	SNV	c.4475C>A	p.Ser1492Tyr	131	611	21.4
22C	*ADAMTS20*	chr12	43825188	SNV	c.3208A>C	p.Ser1070Arg	188	1801	10.4
22C	*ADAMTS20*	chr12	43825231	SNV	c.3165G>C	p.Leu1055Phe	209	1873	11.2
22C	*ERBB3*	chr12	56495511	SNV	c.3701C>G	p.Ser1234Cys	156	857	18.2
22C	*TP53*	chr17	7577120	SNV	c.818G>T	p.Arg273Leu	1212	2947	41.1
22C	*COL1A1*	chr17	48268222	SNV	c.2299G>A	p.Gly767Ser	196	776	25.3
22C	*MYCN*	chr2	16082565	SNV	c.379G>C	p.Glu127Gln	167	1061	15.7
22C	*PIK3CA*	chr3	178952085	SNV	c.3140A>G	p.His1047Arg	500	904	55.3
22C	*PKHD1*	chr6	51524393	SNV	c.10531G>C	p.Gly3511Arg	91	295	30.9
22C	*WRN*	chr8	31024698	SNV	c.4143C>G	p.Ile1381Met	33	276	12.0
22C	*PRKDC*	chr8	48749850	SNV	c.7683C>G	p.Phe2561Leu	191	1407	13.6
23C	*NOTCH2*	chr1	83355^12048	Insertion	c.3005_3006insG	p.Cys1002fs	232	1133	20.5
23C	*TP53*	chr17	7577539	SNV	c.742C>G	p.Arg248Gly	452	1602	28.2
23C	*TAF1L*	chr9	32635415	SNV	c.163A>G	p.Ser55Gly	14	59	23.7
23C	*TAF1L*	chr9	32635418	SNV	c.160G>A	p.Glu54Lys	10	59	17.0
24C	*TP53*	chr17	7577560	SNV	c.721T>C	p.Ser241Pro	108	628	17.2
25C	*TP53*	chr17	7577099	SNV	c.839G>A	p.Arg280Lys	111	375	29.6
25C	*RNF213*	chr17	78363848	SNV	c.15322G>T	p.Asp5108Tyr	158	401	39.4
25C	*PIK3CA*	chr3	178952085	SNV	c.3140A>T	p.His1047Leu	604	903	66.9
27C	*CSMD3*	chr8	113259311	SNV	c.10160G>A	p.Gly3387Glu	64	416	15.4
29C	*TET1*	chr10	70332487	SNV	c.392C>G	p.Ser131Cys	201	383	52.5
29C	*BRD3*	chr9	136901353	SNV	c.1737G>C	p.Gln579His	199	674	29.5
36C	*no mutation*								
38C	*FGFR2*	chr10	123239535	SNV	c.2302G>T	p.Glu768*	1949	1981	98.4
38C	*TP53*	chr17	7578271	SNV	c.578A>G	p.His193Arg	102	162	63.0
39C	*ADAMTS20*	chr12	43886366	SNV	c.1018C>A	p.Gln340Lys	247	1209	20.4
39C	*TNK2*	chr3	195595035	SNV	c.2323C>T	p.Pro775Ser	164	540	30.4
39C	*PIK3CG*	chr7	106509015	SNV	c.1009G>A	p.Glu337Lys	322	979	32.9
40C	*no mutation*								
41C	*PIK3CA*	chr3	178936082	SNV	c.1624G>A	p.Glu542Lys	189	721	26.2
42C	*TP53*	chr17	7578268	SNV	c.581T>G	p.Leu194Arg	56	460	12.2
42C	*PIK3CA*	chr3	178952085	SNV	c.3140A>G	p.His1047Arg	41	312	13.1
44C	*EML4*	chr2	42557216	SNV	c.2815G>A	p.Glu939Lys	311	1880	16.5
45C	*no mutation*								
46C	*ABL2*	chr1	179077451	Deletion	c.2951delC	p.Pro984fs	61	238	25.6
46C	*TP53*	chr17	7577112	SNV	c.826G>C	p.Ala276Pro	133	604	22.0
46C	*PIK3CA*	chr3	178952085	SNV	c.3140A>G	p.His1047Arg	182	455	40.0
49C	*AKT1*	chr14	105246551	SNV	c.49G>A	p.Glu17Lys	3209	15573	20.6
50C	*MAP2K4*	chr17	12016603	SNV	c.739G>T	p.Asp247Tyr	107	342	31.3
50C	*ALK*	chr2	29456486	SNV	c.2432T>A	p.Val811Glu	96	420	22.9
122C	*no mutation*								

Supplementary Table 4 List of candidate genes isolated by expression micorarray

Probe Name	Chromo-some	Gene	Expression level (intensity) in samples with		P-value	Fold (log2)	Abundant expression
			pCR	non-pCR			
A_33_P3260634	chr20	*PLCB4*	1.41	0.06	0.0002	4.5	✓
A_24_P402438	chr1	*TGFB2*	0.87	0.04	0.00029	4.4	✓
A_23_P214267	chr6	*GPR110*	0.54	0.03	0.00034	4.3	✓
A_33_P3221203	chr11	*MMP13*	2.00	0.14	0.00057	3.8	✓
A_23_P259333	chr6	*C6orf203*	2.14	0.19	0.00034	3.5	✓
A_23_P1691	chr11	*MMP1*	5.66	0.66	0.00033	3.1	✓
A_23_P69573	chr4	*GUCY1A3*	2.30	0.29	0.00031	3.0	✓
A_23_P154065	chr2	*TUBA4A*	22.63	4.92	0.00098	2.2	✓
A_33_P3403867	chr20	*PMEPA1*	5.28	1.15	0.00084	2.2	✓
A_33_P3249872	chr22	*FBLN1*	11.31	2.64	0.00003	2.1	✓
A_33_P3263824	chr10	*MRPL43*	0.57	2.46	0.00036	-2.2	✓
A_23_P68121	chr2	*PSD4*	0.03	0.14	0.00025	-2.3	
A_33_P3378514	chr4	*PDE5A*	0.57	4.00	0.00023	-2.7	✓
A_33_P3374289	chr10	*C10orf82*	0.05	0.41	0.00009	-3.2	
A_23_P69699	chr4	*NPY1R*	0.01	0.15	0.00088	-3.8	

Supplementary Table 5 List of candidate genes isolated by DNA methylation beadarray

TargetID	Chromosome	Gene	UCSC RefGene Group	Relation to a CpG Island	Sensitivity	Specificity	Accuracy	Pyrosequencing primer
cg21831898	1	*C1orf51*	TSS1500	Island	0.38	0.95	0.74	Designed
cg26797073	2	*PTPN18*	Body	Island	0.31	0.95	0.71	Designed
cg18925923	4	*LOC441046*	TSS1500	N Shore	0.15	1.00	0.68	Designed
cg15896301	5	*HSD17B4*	1stExon	Island	0.62	0.90	0.79	Designed
cg12623648	5	*ZNF354C*	TSS1500	Island	0.15	1.00	0.68	Failed
cg08496452	10	*TTC18*	TSS200	Island	0.15	1.00	0.68	Designed
cg11934304	15	*IVD*	Body	Island	0.38	0.90	0.71	Designed
cg26448406	22	*PISD*	5'UTR	Island	0.31	0.95	0.71	Failed

Supplementary Table 6 Detailed responses by *HSD17B4* methylation and ER status

HSD17B4		Pathological response								pCR
methylation	ER	0	1a	1b	2a	2b	rDCIS	pCR	Total	rate
High	Negative	0	0	1	2	2	1	24	30	80%
High	Positive	0	3	0	1	0	0	6	10	60%
Low	Negative	0	4	2	4	4	10	11	35	31%
Low	Positive	1	10	12	14	8	6	2	53	4%

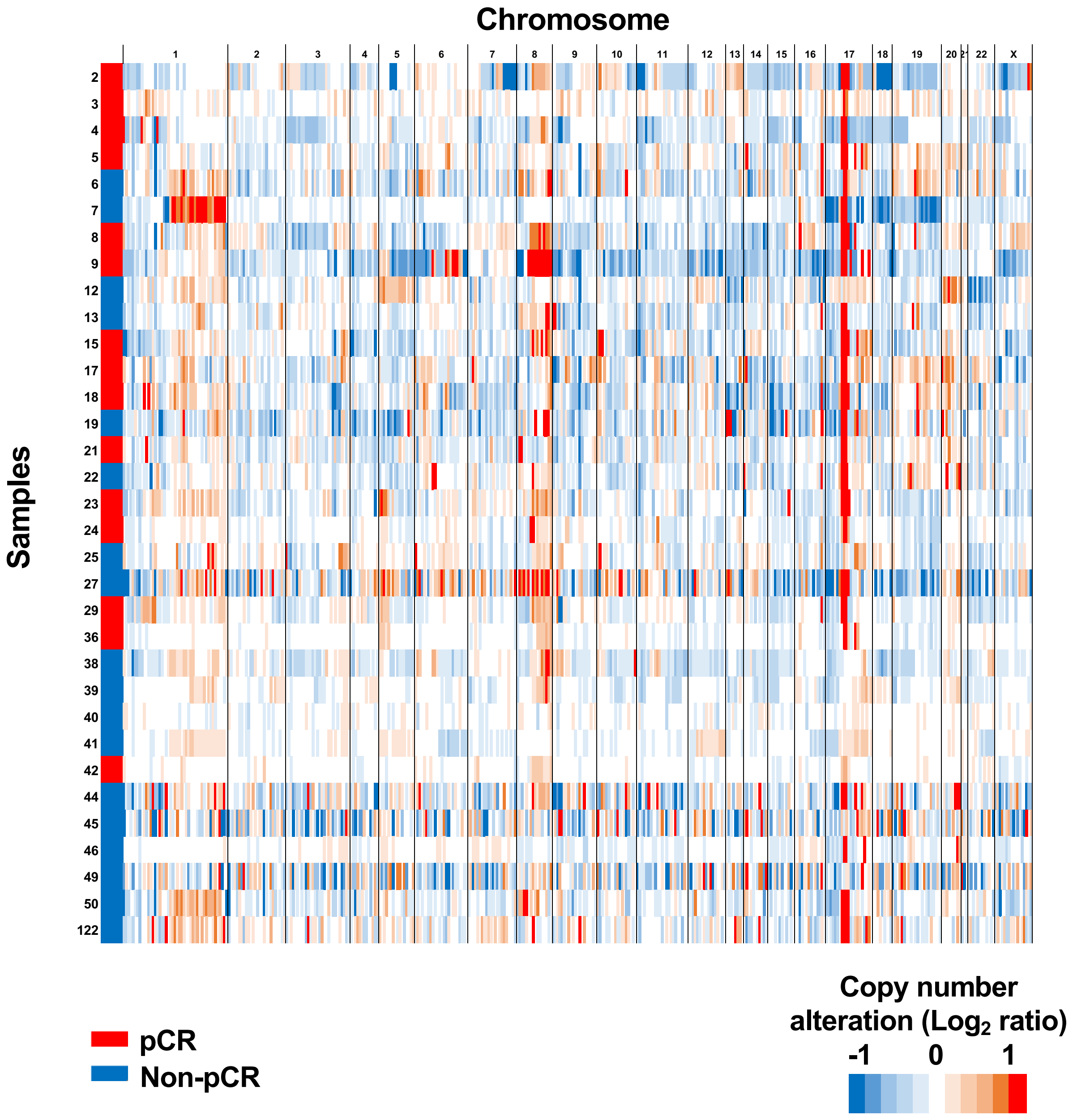

Supplementary Figure 1

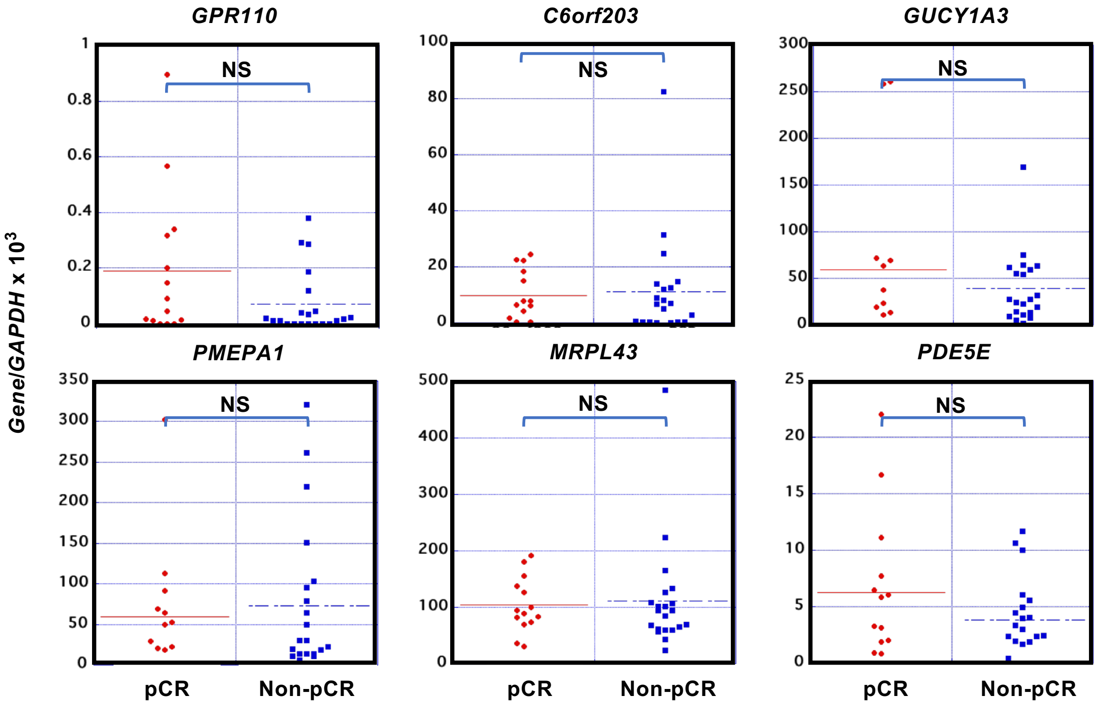

Supplementary Figure 2

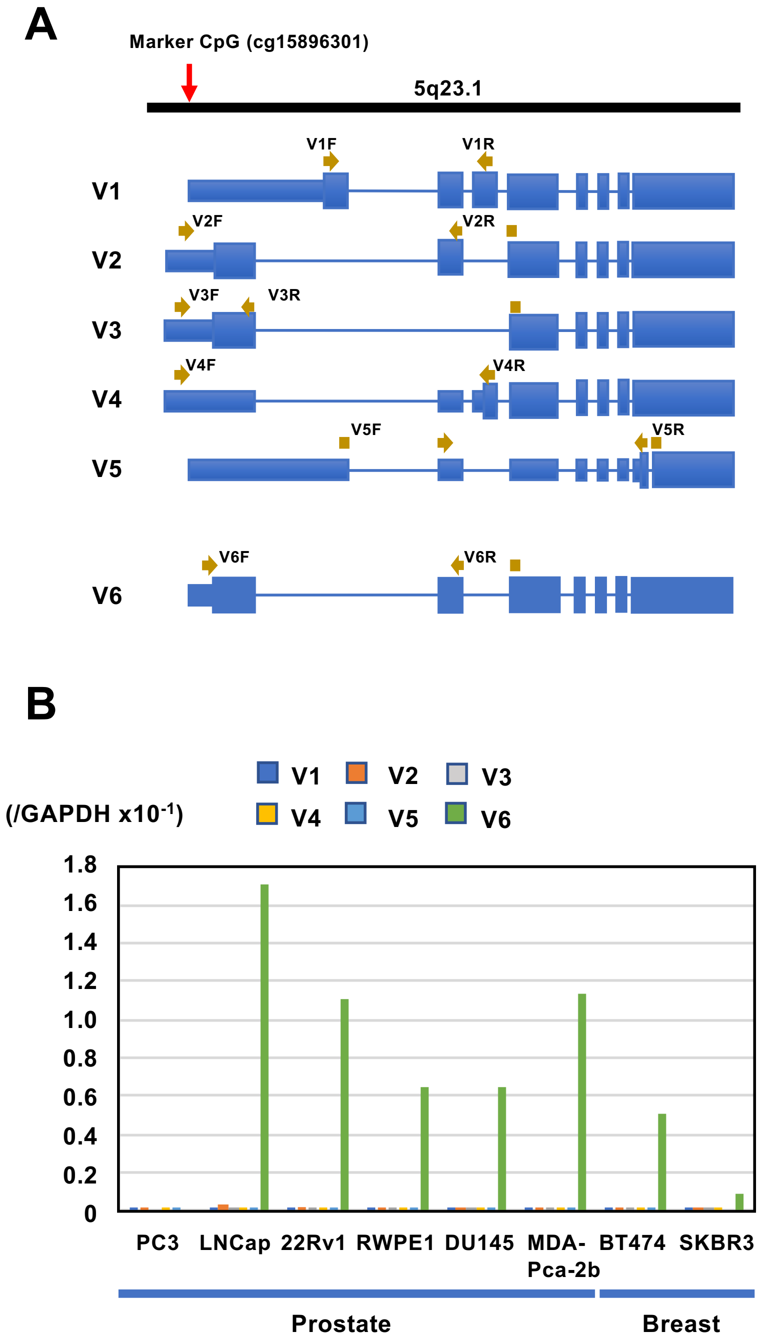

Supplementary Figure 3

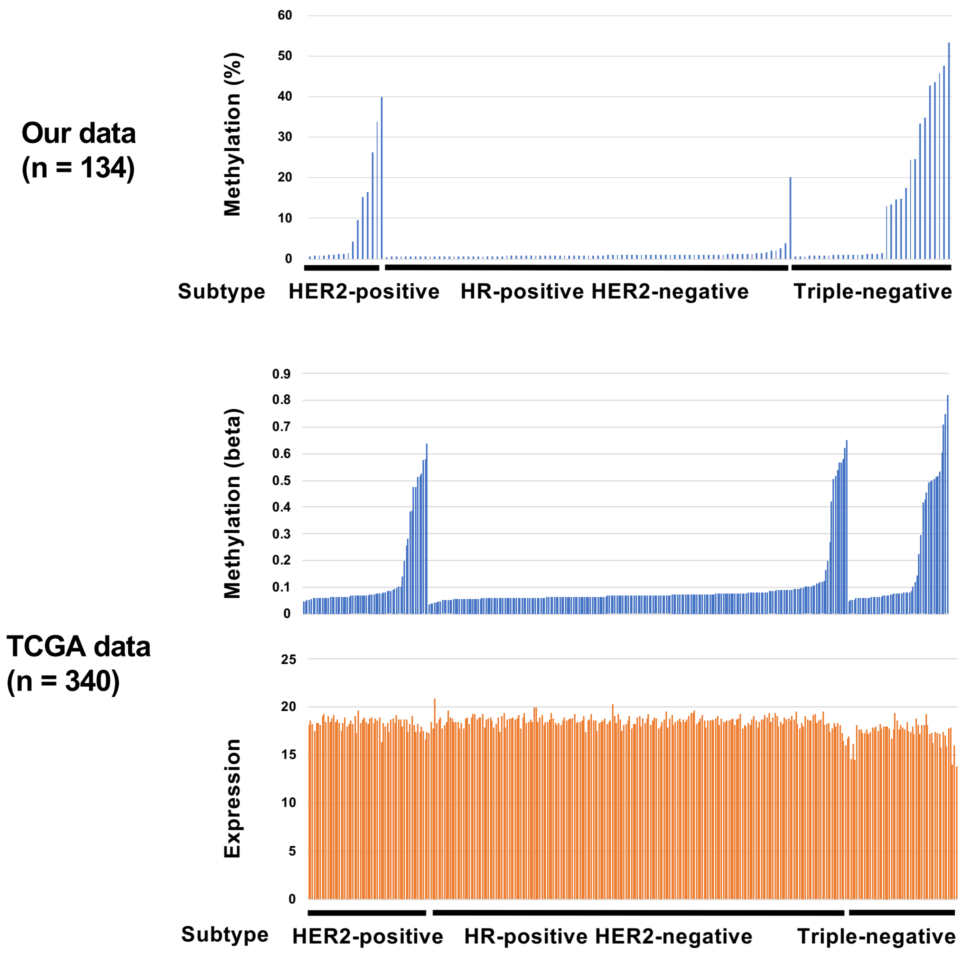

Supplementary Figure 4

Supplementary figure legends

Supplementary Figure 1. Copy number alterations in the screening set.

A heatmap of copy number alterations of 409 genes measured by target sequencing in the screening set.

Supplementary Figure 2. Expression analysis of the remaining 6 genes in the validation set.

Expression levels of the 6 of 12 genes not shown in Figure 2B measured by real-time RT-PCR.

Supplementary Figure 3. Transcriptional variants of HSD17B4

(A) Exon and intron structures of *HSD17B4* transcriptional variants. V1 to V5 had been reported, and V6 was identified in this study. The primers used for real-time RT-PCR are shown by arrows. (B) Expression of *HSD17B4* transcriptional variants in breast and prostate cancer cell lines.

Supplementary Figure 4. HSD17B4 methylation and expression in breast cancer tissues in the TCGA database

HSD17B4 methylation in breast cancer tissues in our specimens (n = 134, upper), *HSD17B4* methylation (middle) and expression (lower) in breast cancer tissues in the TCGA database (n = 340) are shown. Breast cancers with *HSD17B4* methylation were limited to specific subtypes, HER2-positive and triple-negative breast cancers. High *HSD17B4* expression was observed even in breast cancer tissues with its methylation, which was considered due to high *HSD17B4* expression in normal cells contaminating cancer tissues.
